# Syngas Production From the Reforming of Typical Biogas Compositions in an Inert Porous Media Reactor

**DOI:** 10.3389/fchem.2020.00145

**Published:** 2020-03-13

**Authors:** Fabián Guerrero, Lorena Espinoza, Nicolas Ripoll, Pilar Lisbona, Inmaculada Arauzo, Mario Toledo

**Affiliations:** ^1^Department of Mechanical Engineering, Universidad Técnica Federico Santa Maria, Valparaíso, Chile; ^2^Department of Agricultural and Forestry Engineering, Universidad de Valladolid, Valladolid, Spain; ^3^Instituto Universitario Mixto CIRCE, Universidad de Zaragoza, Zaragoza, Spain

**Keywords:** syngas production, reforming, filtration combustion, CO_2_ utilization, biogas

## Abstract

Syngas production by inert porous media combustion of rich biogas–air mixtures was studied experimentally, focusing on carbon dioxide utilization and process efficiency. Different gas mixtures of natural gas and carbon dioxide, which simulated a typical biogas composition of 100:0, 70:30, 55:45, and 40:60 (CH_4_:CO_2_), were comparatively analyzed considering combustion waves temperatures and velocities, and chemical concentrations products, at high equivalence ratios of φ = 1.5 and φ = 2.0. Different CO_2_ concentrations on biogas composition showed higher H_2_ productions than on pure methane (100:0), mainly due to CO_2_ reforming reactions. Also, syngas production, hydrogen yields, and process efficiency by means of biogas filtration combustion were higher than under methane filtration combustion. Results of the thermochemical conversion of biogas show an alternative and promising non-catalytic technique to CO_2_ utilization.

## Introduction

The current climate change scenario has directed special efforts of governmental bodies and policymakers all over the world toward finding alternatives capable of reducing and reversing damage already done to the environment (Moral et al., 2018). The need for a paradigm shift regarding energy production and waste management has forced the research and development of new technological alternatives that contribute to the mitigation of anthropogenic impact while driving to sustainable development. In this context, there is an increasing interest in bioenergy production, because it allows the use of biomass wastes as feedstock for carbon-neutral energy production (Sahota et al., 2018). Bioenergy in the form of biogas can contribute to the reduction of anthropogenic greenhouse gas emissions, mainly produced by combustion of conventional fossil fuels and negligent waste disposal.

Biogas, a gas fuel mixture mostly composed of methane (CH_4_, 40–65% vol/vol) and carbon dioxide (CO_2_, 35–55% vol/vol) with a lower concentration of hydrogen sulfide (H_2_S, 0.1–3.0% vol/vol), water (H_2_O), and other trace compounds (Miltner et al., 2017), has an usual lower heating value in the range of 20 and 25 MJ/m^3^ for CH_4_ contents between 60 and 65% (Angelidaki et al., 2018). It is produced from the decomposition of wet biomass within an oxygen (O_2_) lacking atmosphere, process known as anaerobic digestion (AD). Particularly, this type of biomass with high moisture content represents a relevant fraction of several organic wastes, such as urban waste, as well as food and agricultural industrial waste (Kothari et al., 2010; Achinas et al., 2017). Biogas production has the particularity that suits a variety of biological sources that are available in the form of unwanted materials; thus, it is considered as an accessible and decentralized energy carrier, which has had growing participation in the global energy matrix (Scarlat et al., 2018), representing nowadays 35% of the energy produced from biomass sources (Rasapoor et al., 2020). Its applications consider heat and power generation, both at the domestic level (cooking, H_2_O and space heating) and industrial scale (combined heat and power plants, transportation fuel for vehicles, and electricity generation through fuel cells) (Cozzolino et al., 2017; Kadam and Panwar, 2017; Kim and Sung, 2018; Saadabadi et al., 2019).

Despite the aforementioned applications, the variability in its chemical composition and low heating value of the mixture, besides the presence of undesired compounds in raw biogas, are the most determining factors that limit its range of application and scaling-up (Kadam and Panwar, 2017). Trace amounts of H_2_S, ammonia (NH_3_) and siloxanes in biogas can result in significant harm for any thermal conversion device, as well as distribution and storage facilities, such as corrosion, fouling, and harmful environmental emissions, which increase hazards for human health (Sun et al., 2015). On the other hand, the energy content of biogas depends directly on the CH_4_ share in the mixture. Moreover, waste sources differ significantly, both in their qualities and quantities, depending on the nature of their origin (Sebola et al., 2014); therefore, biogas is subjected to high variability in its composition (Table 1) (Seiffert et al., 2009; Sun et al., 2015; Gao et al., 2018; Izzah et al., 2019; Katinas et al., 2019). In particular, CH_4_ yield depends on the nature of the substrate, pH, climatic conditions, operational temperature, and pressure among others (Kadam and Panwar, 2017; Angelidaki et al., 2018). Additionally, inert gases such as CO_2_ and nitrogen (N_2_), reduce the fuel's energy potential by not participating in the exothermic reactions involved in the combustion process.

**Table 1:** Average composition of raw biogas according to the biomass-substrate source.

**Biomass Substrate**	**CH_**4**_ (%)**	**References**
Landfill	35–65	Sun et al., 2015
Landfill	40–50	Katinas et al., 2019
Landfill	35–65	Gao et al., 2018
Sewage sludge	58–75	Gao et al., 2018
Urban sewage sludge	60–65	Katinas et al., 2019
Agricultural waste	45–75	Gao et al., 2018
Agro food waste	60–70	Katinas et al., 2019
Fruit/vegetable production	56	Seiffert et al., 2009
**Content**	**CH**_**4**_**:CO**_**2**_ **(%)**	**References**
Carbohydrates	50:50	Izzah et al., 2019
Proteins	60:40	Izzah et al., 2019
Fats	70:30	Izzah et al., 2019

Therefore, biogas cleaning and its upgrade to a higher fuel standard are of particular relevance (Sun et al., 2015). Currently, both biogas cleaning and upgrading techniques focus on removal of volatile organic compounds, siloxanes, carbon monoxide (CO), and NH_3_, with primary attention to H_2_S and CO_2_. Available methods include physical absorption, membrane separation, cryogenic separation, and chemical conversion. However, all the aforementioned techniques are energy demanding with medium to high economic cost (Angelidaki et al., 2018).

Conventionally, biogas upgrade involves the removal of CO_2_ in order to obtain high concentrations of biomethane reaching natural gas (NG) standards. However, this approach does not serve as an efficient solution because, in order to suit gas standards for removed CO_2_ applications, it is necessary to invest considerable amounts of energy, as different utilization processes have different requirements regarding CO_2_ concentration on gas quality (Sun et al., 2015). On the other hand, the thermochemical approach of biogas reforming achieves to take an advantage of main compounds found in typical biogas compositions, that is, CH_4_ and CO_2_, by using both for their conversion into a higher density fuel in the form of synthetic gas (syngas), mainly composed of hydrogen (H_2_) and CO.

Thermochemical conversion of biogas in the presence of O_2_ could be modeled as a balance between exothermic and endothermic reactions. Where complete combustion (CC) and partial oxidation (POX) reforming of CH_4_ release heat, capable of sustaining endothermic reactions associated with CO_2_ interaction by dry reforming (DR) mechanism. Thus, an indirect reaction pathway for biogas upgrade would consider CC, POX (Gao et al., 2018), DR, bi-reforming (BR), oxi-CO_2_ reforming (OR), and the reverse H_2_O gas shift reaction (rWGS) (Zeng et al., 2017; Stroud et al., 2018).

(R.1) $$\begin{array}{l} \text{CC}:{\text{CH}}_{4}+2{\text{O}}_{2}↔{\text{CO}}_{2}+2{\text{H}}_{2}\text{O }\Delta H^{o}=-803\text{ kJ}/\text{mol } \end{array}$$

(R.2) $$\begin{array}{l} \text{POX}:{\text{CH}}_{4}+0.5{\text{O}}_{2}↔\text{CO}+{2\text{H}}_{2}\;\Delta H^{o}=-35.6\text{ kJ}/\text{mol } \end{array}$$

(R.3) $$\begin{array}{l} \text{DR}:{\text{CH}}_{4}+\text{C}{\text{O}}_{2}↔2\text{CO}+2{\text{H}}_{2}\;\Delta {\text{H}}_{298\text{K}}=+247\text{ kJ}/\text{mol};\\ \;\Delta G_{298K}=+170\text{ kJ}/\text{mol } \end{array}$$

(R.4) $$\begin{array}{l} \text{BR}:3\text{C}{\text{H}}_{4}+\text{C}{\text{O}}_{2}+2H_{2}O↔4CO+8H_{2}\;\Delta {\text{H}}_{298\text{K}}=+220\text{ kJ}/\text{mol};\\ \;\Delta G_{298K}=+151\text{ kJ}/\text{mol } \end{array}$$

(R.5) $$\begin{array}{l} \text{OR}:3\text{C}{\text{H}}_{4}+\text{C}{\text{O}}_{2}+{\text{O}}_{2}↔4\text{CO}+6{\text{H}}_{2}\;\Delta H_{298\text{K}}=+58\text{ kJ}/\text{mol};\\ \;\Delta G_{298\text{K}}=-1\text{ kJ}/\text{mol } \end{array}$$

(R.6) $$\begin{array}{l} rWGS:{\text{H}}_{2}+\text{C}{\text{O}}_{2}↔\text{CO}+{\text{H}}_{2}O\;\Delta H_{298\text{K}}=+41.1\text{ kJ}/\text{mol} \end{array}$$

Available methods for biogas reforming consider the use of continuous beds, fluidized beds, supercritical H_2_O, and membrane catalytic reactors (Gao et al., 2018; Remón et al., 2018). However, the major drawbacks of these approaches are associated to the decrease in efficiency as consequence of catalytic wearing caused by the elevated temperatures needed for cost-effective biogas reforming into syngas (Jing et al., 2004; Chen et al., 2005; Hou et al., 2007; Gao et al., 2008). Commercial development has also been limited because of the effects of sintering and coking, widely reported by other researchers to occur over 1,100 K, being both phenomena responsible for the precipitation of solid materials driving to catalytic deactivation (Boullosa-Eiras et al., 2011; Moral et al., 2018). Therefore, considering thermal restrictions observed in the catalytic approach, alternative methods base on non-catalytic techniques have been promoted, such as plasma-assisted non-thermal arc discharge (Mao et al., 2018), solar thermal aerosol flow reactors (Gao et al., 2018), and inert porous media (IPM) reactors.

Partial oxidation and DR in IPM, also known as filtration combustion, has been proposed as a feasible alternative to achieve high-temperature non-catalytic reforming of carbonaceous fuels, such as biogas (Zeng et al., 2017) and pure CH_4_ (Drayton et al., 1998; Bingue et al., 2002; Itaya et al., 2002; Smith et al., 2013; Abdul Mujeebu, 2016). The existence of a porous solid structure within the reaction zone promotes the recirculation of heat within the reactor, thus effectively increasing the heat transfer that leads to an excess on the enthalpy of the process (Hardesty and Weinberg, 1973; Kamal and Mohamad, 2006; Al-Hamamre and Al-Zoubi, 2010; Wang et al., 2018). This phenomenon allows the formation of self-sustaining reactions over a large range of equivalence ratios (φ), or fuel–air ratios, resulting in syngas production without the need for any extra heating.

Previous experimental research using biogas and filtration combustion with gaseous and solid fuels exhibited promising results (Espinoza et al., 2018; Gonzalez et al., 2018). Thus, combining processes such as DR with POX in IPM would certainly offer several advantages, such as higher efficiencies to produce syngas, compared with independent processes. However, previous studies had focused on the potential of IPM reactors for biogas conversion by promoting non-catalytic POX, and to the best of our knowledge, no research has been done to test the performance of filtration combustion while varying CH_4_ and CO_2_ concentrations in biogas.

Despite the significant potential and interest in the use of biogas as renewable source of energy, its production is still challenging. Heterogeneity in physicochemical and organic composition of the diverse biomass sources (protein, carbohydrate, and lipid content), added to the multiplicity of operating parameters involved in AD processes, has limited the feasibility of biogas applications, mostly due to the low CH_4_ concentration and yield in the gas mixture (Rasapoor et al., 2020). In this scenario, the study of the applicability of IPM reactors for variable CH_4_ content in biogas reforming represents an interesting alternative for non-catalytic biogas upgrading, which could add flexibility to the composition standards for biogas applications (thermochemical approach) and reduce the requirement of pretreatment techniques for AD optimization, thus favoring biogas share in the energy matrix.

In this context, the present study aims to investigate the impact of CO_2_ in different compositions of biogas [100:0, 70:30, 55:45, and 40:60 (CH_4_:CO_2_)] to evaluate syngas production by the thermal conditions reached by filtration combustion inside an IPM reactor, with a special focus on observing the behavior of the technology under high concentrations of CO_2_ and its impact on DR mechanisms and process efficiency. The experimental parameters used to evaluate the effect of CO_2_ concentration in biogas composition for syngas production were the composition of product gases, reactants conversion (X_CH4_, X_CO2_), product yields (Y_H2_, Y_CO_), reforming efficiency (η_ref_), and H_2_ to CO ratio (H/C).

## Materials and Methods

### Experimental Setup

**Figure 1:**
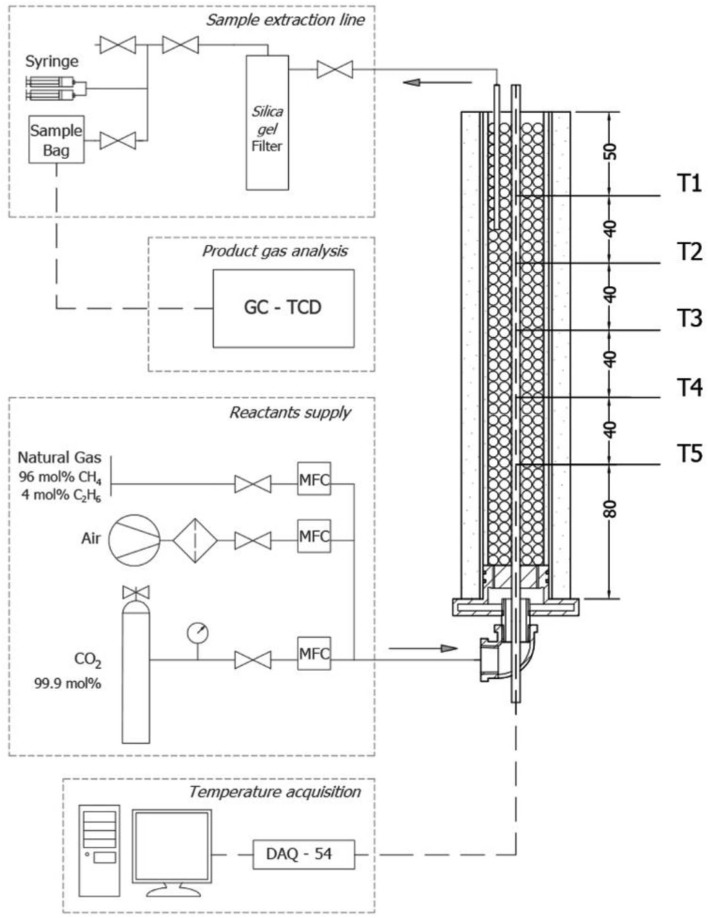
Schematic of the experiment setup.

Filtration combustion experiments with varying fractions of synthetic biogas mixtures with air were conducted using the layout schematically represented in Figure 1. The setup was composed of a reactants supply system, an IPM reactor, a sample extraction line, and a data acquisition system for product gas composition analysis and temperature recording. The reforming of biogas was carried out in a cylindrical tube made of quartz, with a length of 290 mm, a wall thickness of 2 mm, and an inner diameter of 39 mm operating at atmospheric pressure. The reactor was packed with solid alumina spheres (Al_2_O_3_, 5.5 mm diameter), forming a porous matrix with a porosity of ~40%. This represents the void fraction within the cylinder filled with solid spheres, and it was calculated by the quotient between the free volume of pores and the total volume of the solid matrix (Trimis and Durst, 1996). The reactor was insulated with Fiberfrax insulation blankets of 11 and 3 mm thickness in the outer surface and on the inner surface of the quartz tube, respectively.

Fuel supply for biogas mixtures was obtained from the local distribution of NG with a molar concentration of 96% CH_4_/4% C_2_H_6_ on average, whereas CO_2_ was taken from a 99.9 mol% purity Linde Gas S.A cylinder. Dry air was provided by a reciprocating compressor (Qualitas, Miami, FL, USA) operated at room conditions of 20 ± 1°C. Three thermal mass flow controllers (Aalborg GFC17–GFC37; Orangeburg, NY, USA) were implemented for quantitative control of the reactants flows, which were premixed before reaching the inlet at the bottom of the reactor, thus ensuring a homogeneous fuel–air mixture.

Combustion temperature was measured axially along the reactor by 5 type-S thermocouples (Pt/PtRd; OMEGA Engineering Inc., Stamford, CT, USA), with each one located inside a multibore ceramic rod with 6 equally spaced 0.8 mm bores, with a regular spacing on the axis length of 40 mm between junctions (Figure 1, T1–T5); therefore, recorded temperatures are considered to represent the temperature of the solid matrix rather than the gas phase. Data acquisition from each thermocouple signal was achieved with an OMB DAQ-54 A/D module, which sent voltage measurements to a personal computer where signals were translated to temperature readings with the Personal DaqView software (OMEGA Engineering Inc.). An experimental error of 50 K was estimated for temperature measurements.

For product gas analysis, a sample volume of the flue gases was extracted using a ceramic tube of 4.4 mm in diameter located 70 mm inside the Al_2_O_3_ packed bed from the top of the reactor. Sample extraction was obtained by a set of two syringes of 60 mL each, which enabled the manual suction of gas samples from the porous matrix through a hygroscopic filter, filled with 250 mL silica gel spheres (3 to 5 mm diameter), ending in a Tedlar sampling bag (Manufacturer: Sigma-Aldrich, Saint Louis, MO, United States) with polypropylene fitting (1 L) for storing and subsequently gas chromatography (GC) analysis.

### Experimental Procedure

Experiments on filtration combustion of synthetic biogas–air mixtures under equivalence ratios (φ) of 1.5 and 2.0 were carried out for volumetric compositions of 100:0, 70:30, 55:45, and 40:60 (CH_4_:CO_2_). Higher equivalence ratios (over φ =2.5) could not be studied because of instabilities of the combustion wave, which extinguished the combustion front, as reported in previous works by (Drayton et al., 1998; Bingue et al., 2002; Toledo et al., 2009). Preparation of synthetic biogas mixtures was done considering that NG was 100% CH_4_. Natural gas–air flows were set at 0.85 and 5.15 L/min for φ = 1.5, and 1.09 and 4.91 L/min for φ = 2.0, respectively, and kept constant, whereas CO_2_ flow was varied according to biogas composition (CH_4_:CO_2_). Total flow for these mixtures varied from 6.00 to 7.63 L/min associated with filtration velocities between 29.2 and 37.2 cm/s (Table 2). Filtration velocity, referred to as the velocity of the gas flowing through the void fraction of the porous material, was calculated at the reactor's inlet on the total volumetric flow divided by the cross-sectional area weighted by the porosity of the medium. Variation of fuel composition, filtration velocity (*v*_*f*_), and equivalence ratio were studied according to their effect on combustion temperature, wave propagation rate (*v*_*w*_), product gas composition, reactants conversion (X_CH4_, X_CO2_), H_2_ and CO yields (*Y*_*H*_2__, *Y*_*CO*_), H_2_ to CO ratio, and conversion efficiency (η_ref_), according to Equations (1) to (6). Conversion efficiency considered lower heating values of 34, 10, and 12 MJ/m^3^ for CH_4_, H_2_, and CO, respectively. Also, exhaust gases were characterized at 1 bar(a) and 948 K, according to the average experimental temperature of thermocouple installed in the reactor's exit.

**Methane conversion**

(1) $$\begin{array}{l} {\text{X}}_{CH_{4}}\left(\%\right)=\;\left(\left({\;{\dot{m}}_{CH_{4}}}^{in}-{\;{\dot{m}}_{CH_{4}}}^{out}\right)\;/\;{\;{\dot{m}}_{CH_{4}}}^{in}\right)\cdot 100 \end{array}$$

**Carbon dioxide conversion**

(2) $$\begin{array}{l} {\text{X}}_{CO_{2}}\left(\%\right)=\;\left(\left({\;{\dot{m}}_{CO_{2}}}^{in}-{\;{\dot{m}}_{CO_{2}}}^{out}\right)\;/\;{\;{\dot{m}}_{CO_{2}}}^{in}\right)\cdot 100 \end{array}$$

**Hydrogen yield**

(3) $$\begin{array}{l} {\text{Y}}_{H_{2}}(\%)=\;\left((y_{H_{2}}^{\;out}\cdot {\dot{V}}_{tot})\;/\;\left(2\cdot \;{\dot{V}}_{CH_{4}}^{\;in}\right)\right)\cdot 100 \end{array}$$

**Carbon monoxide yield**

(4) $$\begin{array}{l} Y_{CO}\left(\%\right)=\;\left(\;\left({{y_{CO}}^{out}\cdot \dot{V}}_{tot}\;\right)/\;\left({\;{\dot{V}}_{CH_{4}}}^{in}+{\;{\dot{V}}_{CO_{2}}}^{in}\right)\right)\cdot 100 \end{array}$$

**H/C**

(5) $$\begin{array}{l} \text{H}/\text{C }\left(-\right)={\;{\dot{n}}_{H_{2}}}^{out}/{\;{\dot{n}}_{CO}}^{out} \end{array}$$

**Reforming efficiency**

(6) $$\begin{array}{l} \eta_{ref}\left(\%\right)\\ =\left(\;\overset{H_{2},\;CO}{\underset{i}{\sum }}\left({{y_{i}}^{out}\cdot \dot{V}}_{tot}\;\cdot LHV_{i}\right)/\;\left({\;{\dot{V}}_{CH_{4}}}^{in}\cdot LH{V_{CH}}_{4}\right)\right)\cdot 100 \end{array}$$

Combustion mixtures were prepared by a continuous method where fuel and air flows were set by mass flow controllers, premixed, and subsequently injected at the bottom of the reactor. For all experiments, upstream propagation (counterflow displacement in relation to reagents flow) was initiated at the reactor's exit giving way to the preheating of the porous matrix. Once the combustion front was stabilized at the bottom of the reactor (T5, max), adjustments to fuel–air flows were made according to the corresponding equivalence ratio and synthetic biogas composition for each case studied. Temperature data were recorded at regular intervals of time (1 s) in each case, allowing the characterization (direction and magnitude) of combustion wave displacement inside the porous matrix. Combustion wave propagation rate had an estimated error of ~10%. It was measured considering the axial displacement of the combustion front along the reactor over the equidistant thermocouple arrangement; so by identifying the time interval involved in the combustion wave displacement from one acquisition point to another, it is possible to quantify the average propagation rate on the axial length, while the direction, upstream or downstream, was indicated based on the direction of reagents flow, being a positive value associated with a coflowing displacement and a negative value with a counterflow.

**Table 2:** Equivalence ratio, flow rate, and filtration velocity of biogas-air and methane-air mixtures used in the experimental measurements.

**Composition of biogas (% vol/vol)**	**Equivalence ratio (–)**	**Composition of biogas-air mixture (L/min)**			**Filtration velocity (cm/s)**
**(CH_**4**_:CO_**2**_)**	**φ**	**CH_**4**_**	**CO_**2**_**	**Air**	**–**
100:0	1.5	0.85	0.00	5.15	29.2
	2.0	1.09	0.00	4.91	29.2
70:30	1.5	0.85	0.37	5.15	31.0
	2.0	1.09	0.47	4.91	31.5
55:45	1.5	0.85	0.70	5.15	32.6
	2.0	1.09	0.89	4.91	33.6
40:60	1.5	0.85	1.28	5.15	35.5
	2.0	1.09	1.63	4.91	37.2

Gas products were extracted when the combustion front reached 160 mm from the bottom of the reactor (T3, max). Permanent gases such as H_2_, CH_4_, CO, and CO_2_ were identified and quantified by GC (Clarus 500; PerkinElmer, Waltham, Massachusetts, United States) using helium as carrier gas and a thermal conductivity detector (TCD) for analytical quantification.

All experiments were run three times to ensure repeatability.

### Chromatographic Analysis

The concentration of H_2_, CO, CH_4_, and CO_2_ was quantified using a gas chromatographer (Clarus 500; PerkinElmer) mounted with two stainless-steel packed columns [1/8 in outer diameter (O.D.) ×2.1 mm inner diameter (I.D.)] in connection to a TCD. Two position electropneumatic gas sampling valves (10- and 6-port; VICI-Valco Instruments Company Inc., Houston, TX, USA) with a 1 mL sample loop were used in the chromatographic configuration. Separation was made by two porous polymer bead columns, 5 m Hayesep N® 80/100 (Supelco Analytical; Sigma-Aldrich, Saint Louis, Missouri, United States) for compounds with a low molecular weight, and 3 ft Molesieve 5A® 80/100 (Restek) for large-molecule adsorption (Espinoza et al., 2018). Quantification with TCD, which is a universal detector that has good sensitivity, extended linearity, and excellent stability (Hilborn and Monkman, 1975; McNair and Miller, 2008), was carried out based on the difference of thermal conductivity between the mobile phase and the gas to be analyzed (Budiman and Nuryatini, 2015). For data acquisition, interpretation, and chromatogram representation, the software Total Chrome Navigator (PerkinElmer) was used.

**Figure 2:**
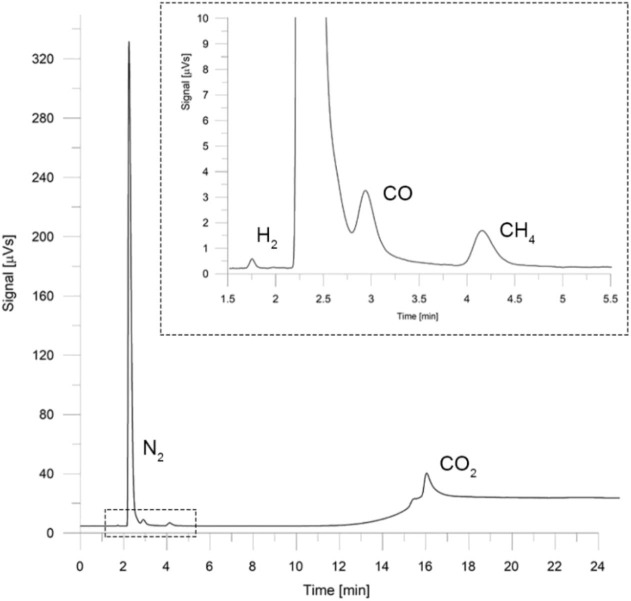
Standard chromatogram used for combustion gas analysis.

As the chromatographic method, splitless injection was established with an injector temperature of 100°C. Helium was used as the carrier gas at a flow rate of 26 mL/min, and the temperature program used in the GC oven was as follows: 35°C initial temperature for 10 min, a temperature rate of 20°C/min until 145°C was reached and held there for 9.50 min. Detector temperature was set at 200°C with a recording frequency of 12.5 pts/s. For subsequent peak identification, the retention time of each compound was obtained as the mean value of 16 sample injections at five different volumes (0.5–2.5 mL in 5 mL increments) of certified standard gas with a 95% confidence level. The results are shown in Table 3. Simple calibration curves were made from the injection of certified standard (Airgas Inc., Radnor Township, Pennsylvania, United States), resulting in correlation coefficients (*r*^2^) between 0.996 and 0.999. The chromatogram presented in Figure 2 shows good selectivity and resolution for peak identification at the retention times reported. An experimental error of 10% was estimated for chemical samples measurements, which considers both the accuracy of laboratory equipment and repeatability of the acquired data.

**Table 3:** Retention time of chemical compounds ($CI=ts/\sqrt{n},C.V=s/x$).

**Compound**	**Retention time (min)**	**C.V (%)**
	$\bar{x}$ **± CI(95%)**	
H_2_	1.73 ± 0.02	2.2
CO	2.85 ± 0.10	3.9
CH_4_	4.12 ± 0.02	1.1
CO_2_	15.76 ± 0.10	1.2

## Experimental Results

Results from experimental syngas production from synthetic biogas reforming, with different biogas–air concentrations, are presented. Four different ratios of CH_4_:CO_2_ were evaluated using the operation conditions shown in Table 2. The comparison of combustion temperature, propagation rate, product gas compositions, reactants conversion, H_2_ yields, H/C ratio, and reforming efficiency is presented for equivalence ratios of 1.5 and 2.0.

### Combustion Wave Propagation Rate and Temperature

**Figure 3:**
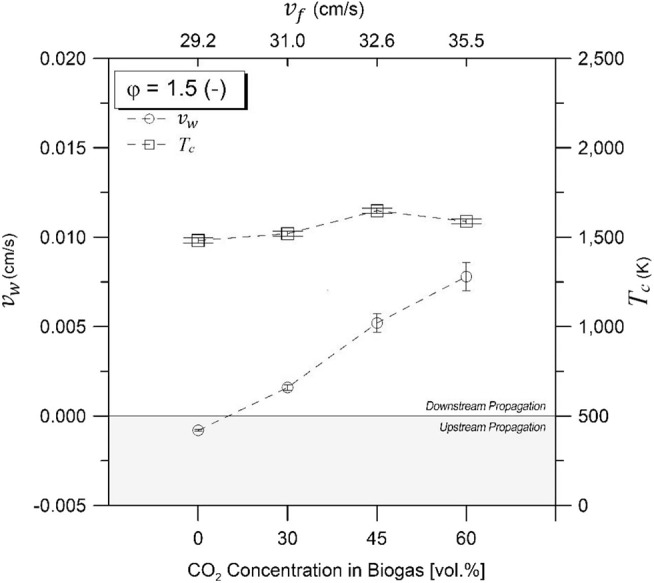
Combustion temperatures and wave velocities for equivalence ratio of φ = 1.5 as function of CO_2_ compositions in biogas. Zero line separates upstream and downstream propagating regions.

**Figure 4:**
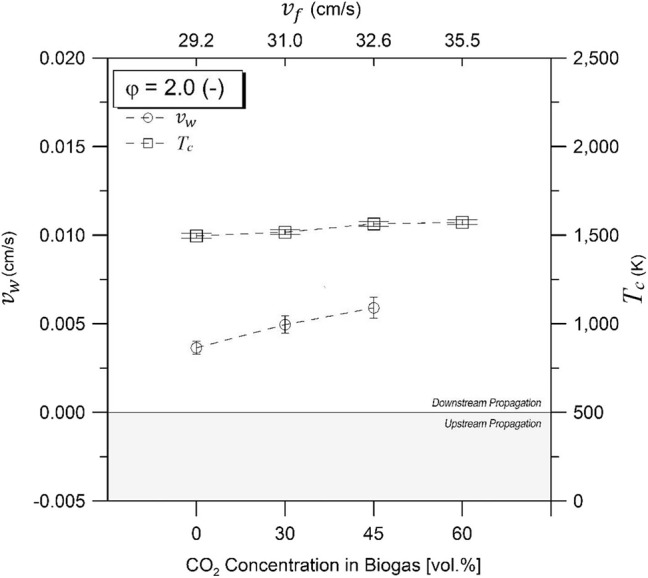
Combustion temperatures and wave velocities for equivalence ratio of φ = 2.0 as function of CO_2_ compositions in biogas. Zero line separates upstream and downstream propagating regions.

Figures 3, 4 illustrate the combustion wave propagation rate and temperature as a function of CO_2_ concentrations in biogas for equivalence ratios of 1.5 and 2.0, respectively. Magnitude of propagation rate (*v*_*w*_) was obtained from experimental temperature profiles, whereas direction of displacement was determined by verifying the sequence of maximum temperatures through thermocouple axial arrangement. Upstream and downstream waves were observed depending on experimental conditions. An enthalpy balance in the filtration combustion wave, without heat losses, demonstrates that the reaction wave propagates upstream for ΔH_g_ <0, and downstream for ΔH_g_ > 0 when a superadiabatic or excess enthalpy flame is formed under the reagent flow direction as reference for displacement characterization inside the porous matrix. Thus, a positive propagation rate indicates that combustion wave displacement occurs in the same direction than reactants flow (coflow, superadiabatic), whereas a negative value refers to a displacement in the opposite direction (counterflow, underadiabatic).

For φ = 1.5 (Figure 3), downstream propagation is observed for 60% vol/vol (maximum absolute value), 45% vol/vol, and 30% vol/vol CO_2_, diminishing its magnitude with a reduction of the filtration velocity and the CO_2_ content in the mixtures. Upstream propagation was exclusively observed at 100% vol/vol CH_4_, whereas for φ = 2.0 (Figure 4), downstream propagation was observed for all experiments tested. For a φ = 2.0 and biogas compositions of 60% vol/vol CO_2_, it was not possible to determine the combustion wave propagation rate with accuracy.

Figures 3, 4 present the maximum experimental temperatures recorded during each test run. Results showed slight differences (<50 K) between φ = 1.5 and φ = 2.0, while maximum combustion temperatures found were 1,564 and 1,563 K for φ = 1.5 and φ = 2.0, respectively, at a biogas composition of 45% CO_2_ in volume. The high combustion temperature with CO_2_ content in the biogas mixtures could be attributed to the role of the exothermic reactions in comparison to the decreasing filtration velocity. It is known that an increasing filtration velocity is responsible for enhancing the diffusion inside the reactor due to larger turbulence inside the pores of the solid matrix (Kennedy et al., 2002). At higher operational temperatures and increasing CO_2_ concentration in the biogas, a higher CO yield is expected as a result of the rWGS reaction, which is a temperature-sensitive endothermic reaction that favors the consumption of H_2_ for CO production.

### Product Compositions

In general, filtration combustion is kinetically controlled. Its products compositions are a function of the combustion wave temperature and residence time. The filtration velocities limit the time available to attain chemical equilibrium in the combustion zone. However, while combustion reactions are relatively fast, the final balance of the reaction products could be controlled by secondary slow reactions (e.g., DR and/or rWGS). The rWGS process could absorb part of the sensible heat and store it in the produced syngas, thus improving the reforming efficiency of the fuel (Zeng et al., 2017).

**Figure 5:**
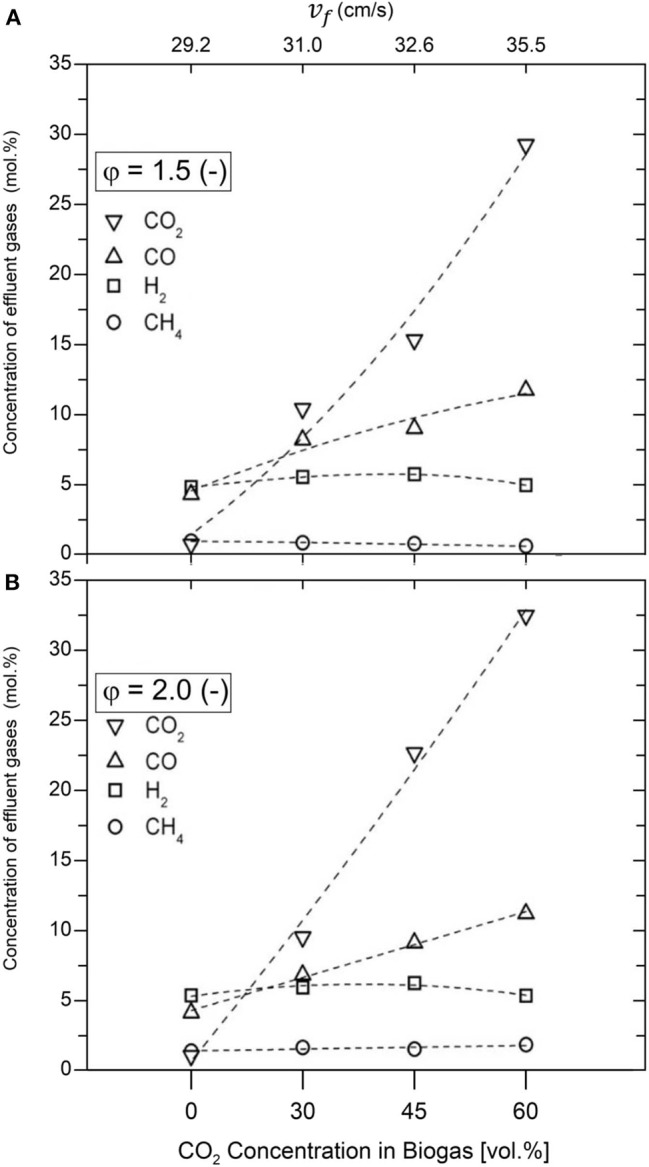
Species concentrations in produced gas (H_2_, CO, CH_4_, and CO_2_) for equivalence ratios φ = 1.5 **(A)** and φ = 2.0 **(B)**.

The molar concentrations of H_2_, CO, CO_2_, and CH_4_ measured during the filtration combustion of biogas–air mixtures are presented in Figure 5. The importance of this information lies in the amount of generated syngas, or specifically, generated H_2_ and the ability of this type of combustion process to promote DR mechanism, thus upgrading biogas proceeding from different feedstock. A minor increase in the concentration of H_2_ (4.82 to 5.32 mol.%) was obtained with varying φ from 1.5 to 2.0, while using a 100% vol/vol CH_4_ composition, which is consistent to previous results, working under similar conditions (Drayton et al., 1998; Bingue et al., 2002; Toledo et al., 2009). Interestingly, the maximum concentrations of H_2_, 6.21 and 5.72 mol.%, were reached with a biogas composition of 45% vol/vol CO_2_, using φ = 2.0 and φ = 1.5, respectively. For biogas composition of 60% vol/vol CO_2_, H_2_ concentration measurements in the product gases were similar to results obtained while operating with pure NG (4.96 and 5.30 mol.%, at φ = 1.5 and φ = 2.0, respectively).

The CO and CO_2_ concentrations in products increased with CO_2_ amount in the biogas mixture, while the concentration of CH_4_ remains almost constant (reaching concentrations below 2.0 mol.%). Because NG/air volumetric flows were kept constant for a given equivalence ratio, CO_2_ injection had no incidence on absolute CH_4_/O_2_ content in the mixture. Therefore, CH_4_ variation in product gases should be attributed to chemical reactions activity as CH_4_ (hydrocarbon fraction in biogas) acts as a limiting reagent in the process. The maximum concentration of CO was 11.74 mol.% using a biogas composition of 60% vol/vol CO_2_, with φ = 1.5, in comparison with 4.30 mol.% obtained using 100% vol/vol CH_4_, while the maximum CO_2_ concentration was 32.47 mol.%, using a biogas composition of 60% vol/vol CO_2_, reached with φ = 2.0, compared with 1.03 mol.% obtained using 100% vol/vol CH_4_. The H_2_ and CO production from biogas might be associated with a combination of CH_4_ POX and DR reactions.

**Figure 6:**
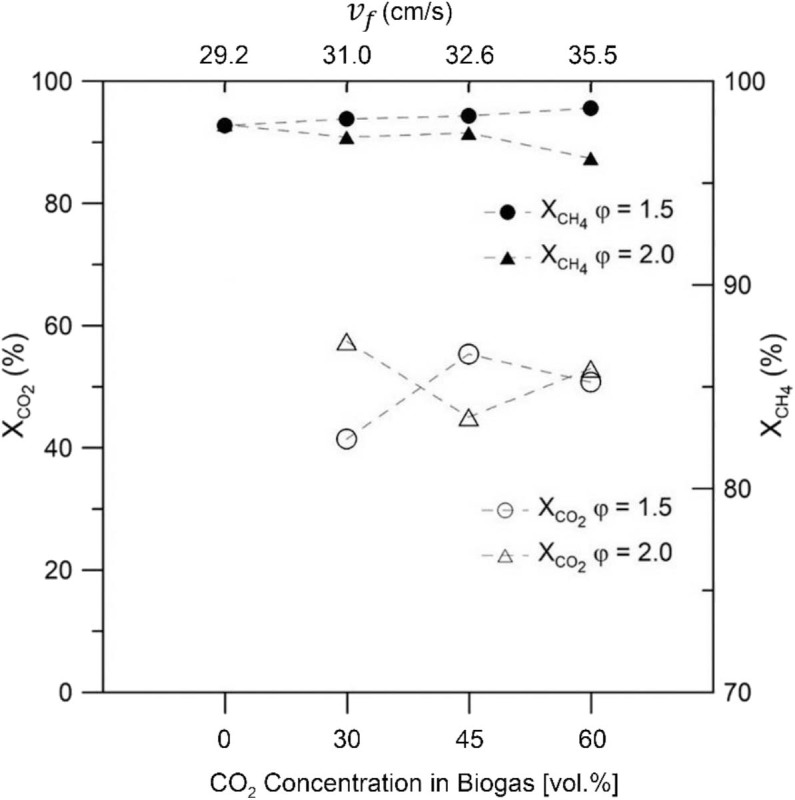
Reactants conversion as function of biogas composition for equivalence ratio of φ = 1.5 and φ = 2.0 as function of biogas mixture.

Figure 6 displays the reactants conversions for both equivalence ratios tested, as function of the CO_2_ content in the synthetic biogas. Results for equivalence ratio of 1.5 showed an increase in CH_4_ conversion as CO_2_ content in reactants was increased, whereas in the case of φ = 2.0, CH_4_ conversion showed a decrease of 1.7% while operating with a biogas composition of 60% vol/vol CO_2_ in comparison to a mixture of 100% vol/vol CH_4_. However, CH_4_ conversion remained >96% over the entire biogas mixture range. Carbon dioxide shows conversions in the range of 40–60% for both equivalence ratios tested.

**Figure 7:**
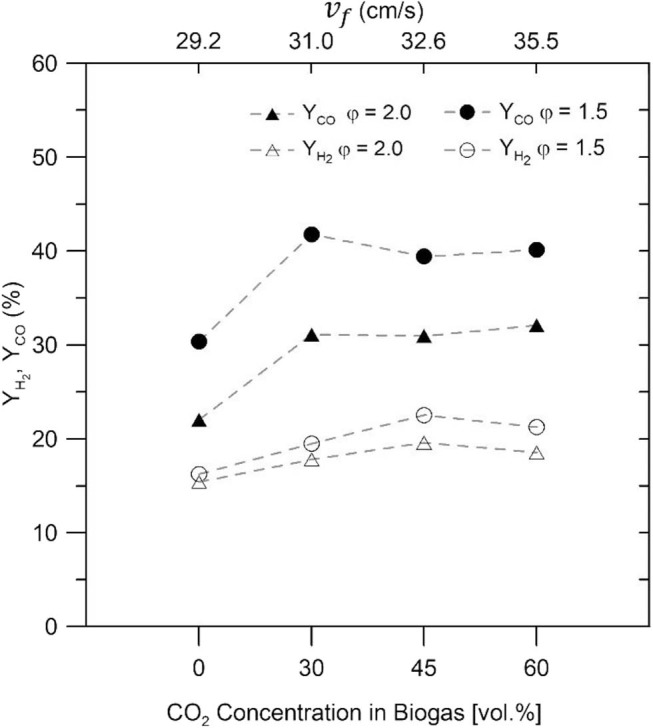
Hydrogen and CO yields for different biogas–air mixtures, and equivalence ratios of φ = 1.5 and φ = 2.0.

Figure 7 illustrates the H_2_ and CO yields (*Y*_*H*_2__ and *Y*_*CO*_) for the biogas–air mixtures in the inert bed, which were determined using Equations (3) and (4). The maximum H_2_ yields recorded for the IPM were 17.68 and 15.3% for φ = 1.5 and φ = 2.0, respectively using only CH_4_. On the other hand, the maximum peaks of H_2_ yields using biogas–air mixtures (at 45% vol/vol CO_2_) in the IPM were 23.34 and 20.4% to φ = 1.5 and φ = 2.0, respectively, before gradually declining with the biogas mixture of 60% vol/vol CO_2_. For different biogas–air mixtures, the CO yields show ~30 and ~40% for φ = 1.5 and φ = 2.0, respectively, but for NG–air mixtures, a decrease in its yield was observed.

**Figure 8:**
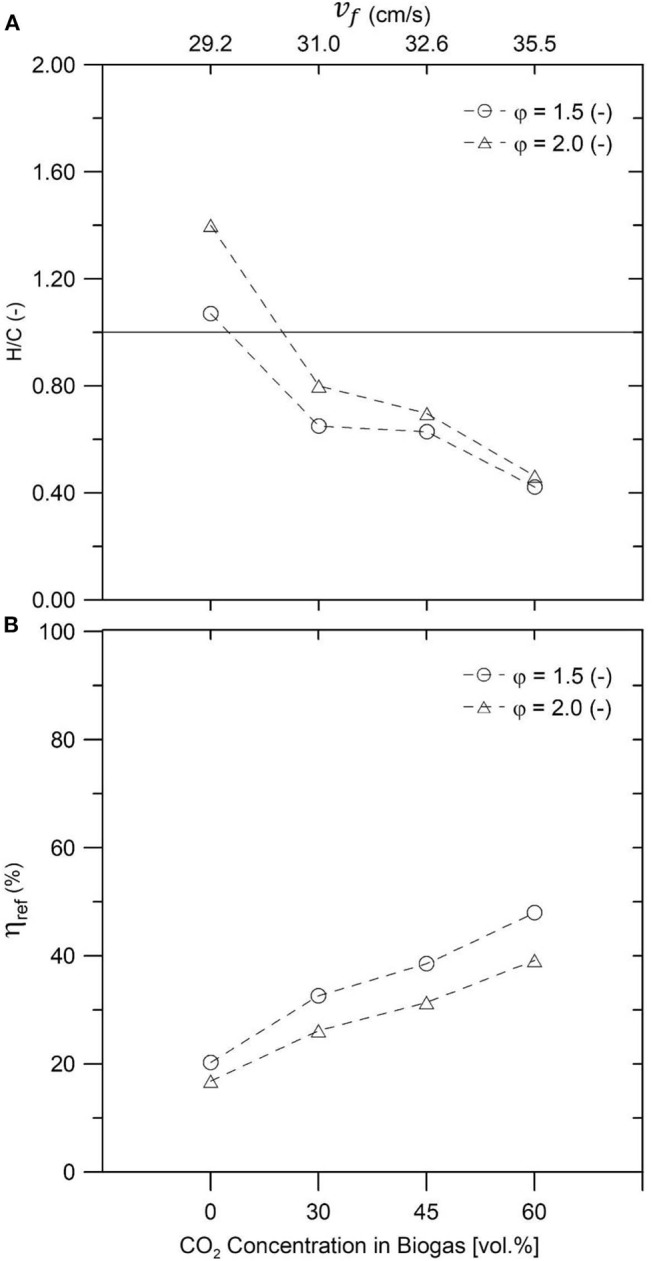
Hydrogen-to-CO ratio **(A)** and reforming efficiency **(B)** for different biogas–air mixtures and equivalence ratios φ = 1.5 and φ = 2.0.

Figure 8A displays H/C ratio of the obtained syngas, portraying a clear increment of the presence of H_2_ in comparison to CO for a decreasing fraction of CO_2_ in the synthetic biogas. This could be related to a shift of the dominant reaction pathway from a mixture of secondary reactions where the rWGS could explain the lower H/C ratios for biogas mixtures with more CO_2_, toward a complete DR (with φ = 1.5 and no CO_2_) and finally reaching a clear POX at φ = 2.0 and no CO_2_. This trend has been previously reported by Zeng et al. (2017) while operating an IPM reactor in a stationary regime with a 50:50 CH_4_:CO_2_ ratio and a filtration velocity of 25.6 cm/s and was attributed to a change of the dominant reaction mechanisms, from DR coupled to the rWGS (with an increased CO_2_ presence in the inlet) to POX (in pure NG conditions).

On the other hand, Figure 8B presents the reforming efficiency of the process as a function of the syngas composition, where a downward tendency is evidenced when the CO_2_ content of the biogas is reduced. Zeng et al. (2017) linked this phenomenon to a considerable amount of sensible energy, released by POX of NG, leaving the reactor through the gaseous products, and the rest being stored as chemical energy in the resulting syngas. However, when the CO_2_ presence was increased, the reforming efficiency was improved due to a greater role of secondary reactions, which, because of the high temperatures from the porous matrix, used the available CO_2_ as a reactant to reform the CH_4_ and produce syngas.

## Conclusions

Carbon dioxide utilization for H_2_ and syngas production by means of filtration combustion waves in an IPM was experimentally studied for rich biogas–air mixtures at equivalence ratios of 1.5 and 2.0. The effect of different compositions of CH_4_ and CO_2_ on biogas was evaluated by CO_2_ addition in the mixtures.

The main analyses of this study are as follows:

(1) Downstream wave propagation was observed for all experiments for biogas–air mixtures. Upstream propagation was exclusively observed at 100:0 (CH_4_:CO_2_) with a φ = 1.5.
(2) High temperatures reached (>1,500 K), which are attributed to the exothermic reactions, favored CO_2_ reforming and generated considerable concentrations of H_2_ and CO.
(3) The injection of CO_2_ on NG–air mixtures increased the fuel-reforming efficiency of the process.

These results prove to be a significant contribution toward the field of thermochemical conversion of alternative fuels (such as biogas) and a promising technique for CO_2_ utilization under a non-catalytic approach.

## Data Availability Statement

The datasets generated for this study are available on request to the corresponding author.

## Author Contributions

FG, LE, NR, and MT substantial contributions to the conception and design of the work. PL and IA analysis and interpretation of data for the work.

### Conflict of Interest

The authors declare that the research was conducted in the absence of any commercial or financial relationships that could be construed as a potential conflict of interest.

## Figures and Tables

**Table:** 

**Symbol**	**Unit**	**Definition**
φ	(—)	Equivalence ratio
*v*_*f*_	(cm/s)	Filtration velocity
*v*_*w*_	(cm/s)	Combustion wave propagation rate
*T_c_*	(K)	Combustion temperature
*y_i_*	(—)	Volumetric fraction of species *i*
*ṅ*_*i*_	(mol/min)	Molar flow rate of species *i*
${\dot{V}}_{i}$	(L/min)	Volumetric flow rate of species *i*
*X_CH4_*	(%)	Methane conversion
*X_CO2_*	(%)	Carbon dioxide conversion
*Y_H2_*	(%)	Hydrogen yield
*Y_CO_*	(%)	Carbon monoxide yield
*H/C*	(—)	Hydrogen–to–carbon monoxide ratio
η_ref_	(%)	Reforming efficiency
